# Common *TLR1* Genetic Variation Is Not Associated with Death from Melioidosis, a Common Cause of Sepsis in Rural Thailand

**DOI:** 10.1371/journal.pone.0083285

**Published:** 2014-01-02

**Authors:** Narisara Chantratita, Sarunporn Tandhavanant, Nicolle D. Myers, Wirongrong Chierakul, Vanaporn Wuthiekanun, Weera Mahavanakul, Direk Limmathurotsakul, Sharon J. Peacock, T. Eoin West

**Affiliations:** 1 Department of Microbiology and Immunology, Faculty of Tropical Medicine, Mahidol University, Bangkok, Thailand; 2 Mahidol-Oxford Tropical Medicine Research Unit, Faculty of Tropical Medicine, Mahidol University, Bangkok, Thailand; 3 Division of Pulmonary and Critical Care Medicine, Department of Medicine, University of Washington, Seattle, Washington, United States of America; 4 Department of Clinical Tropical Medicine, Faculty of Tropical Medicine, Mahidol University, Bangkok, Thailand; 5 Department of Medicine, Sappasithiprasong Hospital, Ubon Ratchathani, Thailand; 6 Department of Tropical Hygiene, Faculty of Tropical Medicine, Mahidol University, Bangkok, Thailand; 7 Department of Medicine, University of Cambridge, Addenbrooke's Hospital, Cambridge, United Kingdom; 8 International Respiratory and Severe Illness Center, University of Washington, Seattle, Washington, United States of America; South Texas Veterans Health Care System and University Health Science Center San Antonio, United States of America

## Abstract

Melioidosis, infection caused by the Gram-negative bacterium *Burkholderia pseudomallei*, is a common cause of sepsis in northeast Thailand. In white North Americans, common functional genetic variation in *TLR1* is associated with organ failure and death from sepsis. We hypothesized that *TLR1* variants would be associated with outcomes in Thais with melioidosis. We collated the global frequencies of three *TLR1* variants that are common in white North American populations: rs5743551 (-7202A/G), rs4833095 (742A/G), and rs5743618 (1804G/T). We noted a reversal of the minor allele from white North American subjects to Asian populations that was particularly pronounced for rs5743618. In the Utah residents of European ancestry, the frequency of the rs5743618 T allele was 17% whereas in Vietnamese subjects the frequency was >99%. We conducted a genetic association study in 427 patients with melioidosis to determine the association of *TLR1* variation with organ failure or death. We genotyped rs5743551 and rs4833095. The variants were in high linkage disequilibrium but neither variant was associated with organ failure or in-hospital death. In 300 healthy Thai individuals we further tested the association of *TLR1* variation with ex vivo blood responses to Pam3CSK4, a TLR1 agonist. Neither variant was robustly associated with blood cytokine responses induced by Pam3CSK4. We identified additional common variation in *TLR1* by searching public databases and the published literature and screened three additional *TLR1* variants for associations with Pam3CSK4-induced responses but found none. We conclude that the genetic architecture of *TLR1* variation differs substantially in southeast Asians compared to other populations and common variation in *TLR1* in Thais is not associated with outcome from melioidosis or with altered blood responses to Pam3CSK4. Our findings highlight the need for additional studies of *TLR1* and other innate immune genetic modulators of the inflammatory host response and determinants of sepsis in southeast Asian populations.

## Introduction

The global burden of sepsis is estimated at up to 19 million cases per year [1]. Much of this burden occurs in low resource settings where limited data suggest that outcomes are particularly poor [2]. In northeast Thailand, melioidosis - infection with the Gram-negative bacterium *Burkholderia pseudomallei* - is the second most common cause of bacteremia and a frequent cause of sepsis [3], [4]. In this setting and despite appropriate antimicrobial therapy, melioidosis mortality is 43% [5]. Melioidosis is endemic in southeast Asia and northern Australia but increasingly found elsewhere in the tropics [3]. As a systemic infection characterized by an inflammatory host response and poor outcomes, melioidosis serves as an informative example of severe Gram-negative sepsis [6]–[10].

Genetic variation accounts for susceptibility to and outcome from infectious disease, and provides a window into mechanisms that underlie the complex pathophysiology of sepsis [11], [12]. Innate immune signaling pathways that titrate the host inflammatory response are of particular interest. Toll-like receptors (TLRs) comprise a subset of innate immune sensors within the IL-1 receptor family [13]. TLR4 is the best-known TLR, initiating an inflammatory cascade in response to ligation of endotoxin (lipopolysaccharide) expressed by Gram-negative bacteria. We have previously examined innate immune host genetic variants that are associated with susceptibility to melioidosis, and found that *TLR4* variants are associated with infection [14]. TLR5 is a bacterial flagellin sensor; we have also shown that a common genetic variant in *TLR5* is highly associated with survival in individuals with melioidosis [15].

TLR1 is another sensor in the TLR family that forms a heterodimer with TLR2 and facilitates innate immune activation upon ligation of bacterial cell wall components such as lipopeptides, peptidoglycan, and lipotechoic acid [13]. Three *TLR1* variants have been described as associated with sepsis, although the relationship is complex. rs5743551 (-7202A/G) is upstream of *TLR1*. In white North American subjects, this polymorphism tags two non-synonymous polymorphisms: rs5743618 and rs4833095 [16]. rs5743618 (1804G/T or 1805G/T), located in the trans-membrane domain of *TLR1*, changes a serine to an isoleucine at amino acid 602. The T allele has been demonstrated by multiple authors to be relatively hypermorphic [16]–[19]. rs4833095 (742A/G or 743A/G) is situated in the extracellular domain and replaces asparagine with serine at amino acid 248. Two studies have shown an association of rs5743551 and rs5743618 with mortality from sepsis in white medical/surgical and trauma patients in Washington, USA or British Columbia, Canada [16], [20]. Similar associations have been demonstrated for organ failure in sepsis patients in Spain and in white medical/surgical patients in the US [16], [21]. Homozygous carriers of the rs5743551 G allele have a higher mortality in sepsis, and, interestingly, the mortality association is stronger for the tag SNP rs5743551 G allele than for the coding rs5743618 T allele [16], [20]. Moreover, the rs4833095 G allele, but not the rs5743618 T allele, is associated with mortality from Gram-positive trauma-related sepsis [20], and the rs4833095 G allele has been associated with infections such as leprosy and malaria in populations where the rs5743618 T allele is infrequent [22], [23], suggesting an independent effect of this variant. The allele frequency of these *TLR1* variants is markedly different around the world [24], potentially leading to differential associations with outcomes from sepsis.

*B. pseudomallei* is recognized by TLR2/1, inducing rapid upregulation of the innate immune response [25]. It is conceivable that functional variation in *TLR1* may modulate host defense in melioidosis. In light of the data showing an important role for TLR1 in human sepsis and as a trigger for *B. pseudomallei*-induced immune system activation, we undertook an analysis of the association of common hypermorphic *TLR1* genetic variants with outcome in a cohort of Thai subjects with culture-proven melioidosis. We also analyzed the association of *TLR1* variants with blood cytokine responses to a TLR1 agonist in healthy Thai subjects. We hypothesized that hypermorphic *TLR1* variation would be associated with altered outcome, including death and organ failure. Notably, we found that the genetic architecture of functional *TLR1* variation is substantially different in southeast Asian populations, that common *TLR1* variation in Thais is not hypermorphic, and that common variation in *TLR1* in Thais is not associated with outcome in melioidosis. Our data suggest that immunogenetic determinants of outcome from Gram-negative infection in Thais differ from previously described determinants in white North American subjects with sepsis.

## Materials and Methods

### Human studies

#### Melioidosis cohort

Subjects with melioidosis were identified among inpatients at Sappasithiprasong Hospital, Ubon Ratchathani, northeast Thailand from 1999 to 2005. A study team screening patients cultured blood, urine, and other relevant samples (e.g. abscess aspirates) for *B. pseudomallei* [26]. Melioidosis status was defined by a positive culture for *B. pseudomallei* from a sample collected by the study team or independently by hospital clinicians. Demographic and clinical data from enrolment until discharge from hospital was recorded by the study team. All patients included in this analysis were Thai. Outcomes for this study were organ failure or death. Organ failure was defined as respiratory failure or shock. The definition for respiratory failure was hypoxia (PaO2 <60 mmHg) or hypercapnia (PaCO2 >50 mmHg) in conjunction with acidaemia (blood pH <7.30) or requirement for mechanical ventilation. The definition of shock was hypotension (systolic arterial blood pressure <90 mmHg, or 40 mmHg lower than patient's normal blood pressure) despite adequate fluid resuscitation or need for vasopressors to maintain systolic blood pressure > = 90 mmHg or mean arterial pressure > = 70 mmHg. Death was defined as in-hospital death or discharge in a moribund condition for palliative care at home. Written informed consent for enrollment into clinical studies of melioidosis was obtained from subjects or their representatives at the time of recruitment.

#### Immuno-assay studies

Three-hundred healthy subjects donating blood at the blood donation center at Sappasithiprasong Hospital in 2010 were recruited for participation as previously described [15] [Chantratita et al, in press]. Subjects were included if they indicated they were Thai and between the ages of 18 and 60 and did not report any history of immunodeficiency or inflammatory conditions, chronic diseases, pregnancy in the past six months, anti-inflammatory medication use in the past week, antibiotic use in the past month, vaccination in the past six months, heavy exercise or alcohol consumption in the past 24 h, or smoking in the past month. All subjects were born in the northeast region of Thailand. Enrolled subjects gave written informed consent to participate and provided a post-donation blood sample.

The Ethical Review Committee for Research in Human Subjects, Ministry of Public Health, Thailand, and the Ethics Committee of the Faculty of Tropical Medicine, Mahidol University, Bangkok, Thailand, and the University of Washington Human Subjects Division Institutional Review Board approved the consent procedure and protocol of these studies.

### Immuno-assays

Three hundred and eighty microliters of fresh whole blood in citrate mixed 1∶1 with RPMI media was added to pre-prepared plates containing 20 µL of Pam3CSK4 (Invivogen, San Diego, CA) for a final concentration of 100 ng/mL. Pam3CSK4 is a specific agonist for TLR2/1. Plates were incubated at 37°C on a shaking incubator under 5% CO_2_ for 6 h before being spun down and plasma removed and frozen at −80°C. IL-6, IL-8, TNF-α, IL-10, MCP-1, IL-1ra, G-CSF, and IL-1β were later assayed in duplicate on a multiplex bead system (Luminex, Austin, TX) using reagents from R&D Systems (Minneapolis, MN). A complete blood count with differential was performed in the hospital clinical laboratory for each subject at the time of phlebotomy.

### Genotyping

DNA was extracted from whole blood of patients with melioidosis using Nucleon BACC3 kits (GE Healthcare, Buckinghamshire, UK) or from whole blood of healthy donors using the QIAamp DNA Blood Midi Kit (Qiagen, Hilden, Germany). We selected *TLR1* polymorphisms rs5743551 (-7202A/G) and rs4833095 (742A/G) to genotype in the melioidosis cohort using an allele-specific primer extension method (Sequenom, Inc.) with reads by a MALDI-TOF mass spectrometer [14], [15] and in the healthy blood donors using Fluidigm SNPtype assays on a Biomark microfluidics real time PCR system. Additional *TLR1* variants were identified as follows: SNP selection of non-synonymous *TLR1* coding variants was performed by searching the HapMap project database (http://hapmap.ncbi.nlm.nih.gov) and based on functional prediction using a FastSNP analysis (http://fastsnp.ibms.sinica.edu.tw) [27]. Tag *TLR1* SNPs were selected from the Han Chinese in Beijing and Japanese in Tokyo populations in the HapMap database for variants with a minor allele frequency (MAF) at least 2% using the HapMap tag-SNP picker option. Genotyping was performed using Fluidigm SNPtype assays on the Biomark real time PCR system.

### Statistical analysis

Deviation from Hardy-Weinberg equilibrium was calculated for each variant using the exact test. In the melioidosis cohort, the power to detect an association between genotype and death was estimated to be 99% for a risk allele frequency of 0.45-0.55, assuming a recessive model and genotype relative risk of 2, 40% mortality in the population, 100 nonsurvivors, 300 survivors, and alpha  = 0.05 [28]. The crude association between genotype or allele count and outcome was performed using the Chi square test. The adjusted analysis was performed using logistic regression, assuming a recessive genetic model, adjusting for age, gender, pre-existing condition, and clinical presentation. The inclusion in the models of *TLR5* variant rs5744168 (1174C/T) that we have previously shown to be associated with outcome from melioidosis [15] did not appreciably alter the *TLR1* genotype associations. Survival analyses were performed with the log rank test. Healthy subject plasma cytokine levels were normalized to monocyte count and log_10_ transformed before analysis by linear regression, adjusting for age, gender, and batch. Analyses were performed with Stata version 11.2 (College Station, Texas). P values ≤0.05 were considered significant.

## Results

### Global variation in common *TLR1* polymorphisms

We first examined data on the global frequency of three well described *TLR1* variants (rs5743551, rs4833095, and rs5743618) in the published literature or publically accessible databases (Table S1) [14], [16], [17], [20], [21], [24], [29]–[32]. Each variant has been associated with susceptibility to infection or outcome from sepsis. We found marked differences in allele frequencies across populations. For each variant there was a general trend of reversal of the minor allele from North American subjects (who were white or of European ancestry) to Asian populations, which was most pronounced for rs5743618. In the CEU population (Utah residents of European ancestry), the frequency of the rs5743618 T allele was 17%. In contrast, in Vietnamese subjects, the frequency of this allele was >99%. The frequency of the rs5743551 G allele was 18% in CEU subjects but 52% in subjects from northeast Thailand. Similarly, the frequency of the rs4833095 G allele was 18% in CEU subjects but 53% in subjects from northeast Thailand.

### rs5743551 or rs4833095 are not associated with outcome in melioidosis

**Figure 1:**
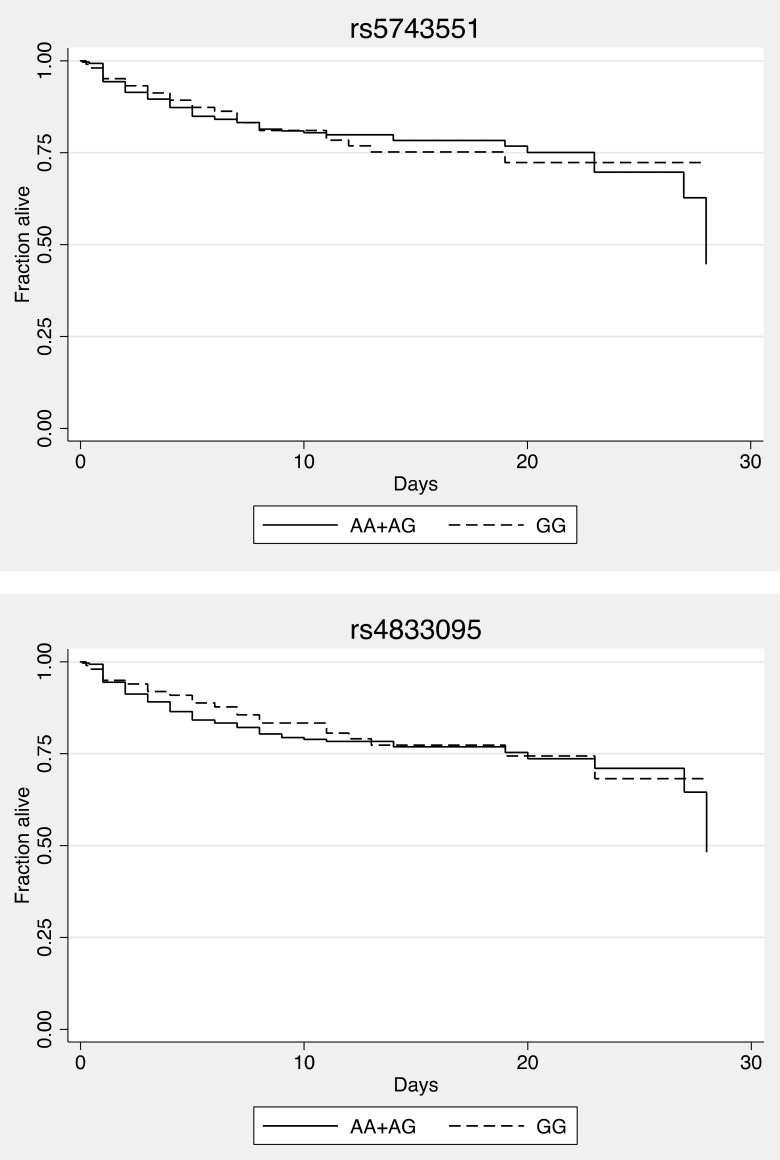
rs5743551 and rs4833095 are not associated with altered survival in melioidosis. For each variant, Kaplan-Meier in-hospital survival curves are plotted for melioidosis patients, grouped by genotype. Curves are not significantly different by the logrank test (P>0.05).

Given the different distribution of *TLR1* alleles in Asia, it is not clear what the associations of these variants may be with outcomes from sepsis in a southeast Asian population. Consequently, we tested whether there was an association between *TLR1* polymorphisms and death or organ failure in 427 Thai subjects with culture proven melioidosis admitted to Sappasithiprasong Hospital, Ubon Ratchathani, Thailand. We have previously reported on TLR variants in these and other subjects at this site [14], [15]. All individuals had culture-proven melioidosis and half were bacteremic. The median age was 49 (IQR 39–60) and 48% were female. The patients presented with bacteremia (50%), pneumonia (40%) and urinary tract infection (12%). Pre-existing conditions were diabetes (55%), chronic liver disease (1%), and kidney disease (6%). Thirty percent developed organ failure, defined as respiratory failure (17%) or shock (24%), and 23% died. We genotyped rs5743551 and rs4833095 but did not genotype rs5743618 as the minor allele (G) frequency was ∼1% in east Asian populations, suggesting that our power to detect an effect would be low. For both variants, we determined that there was no deviation from Hardy-Weinberg equilibrium in the survivors. The variants were in high linkage disequilibrium (R^2^ = 0.79). Following the genetic models described by Wurfel and Thompson [16], [20], we tested the association of homozygous carriage of the rs5743551 G allele with in-hospital death from melioidosis (Table 1) but found no significant effect (adjusted OR 0.99, 95% CI: 0.58–1.71, p = 0.98). Similarly, we found no association with death of homozygous carriage of the rs4833095 G allele (adjusted OR 0.94, 95% CI 0.54–1.63, p = 0.82). Survival curves showed no difference based on genotype (Figure 1). There was no association of either variant with organ failure (Table 1).

**Table 1:** *TLR1* variant genotype and allele frequencies and associations with death in melioidosis.

Marker	Outcome^a^		HWE^b^	Crude^c^	Adjusted^d^		
			P	P	OR	95% CI	P
	Death						
rs5743551	No	Yes					
AA	86 (26.2)	21 (21.9)					
AG	151 (46.0)	48 (50.0)		0.67	0.99	0.58–1.71	0.98
GG	91 (27.7)	27 (28.1)					
			0.15				
A	323 (49.2)	90 (46.9)		0.57			
G	333 (50.8)	102 (53.1)					
rs4833095							
GG	86 (26.2)	25 (25.5)					
AG	167 (50.9)	51 (52.0)		0.98	0.94	0.54–1.63	0.82
AA	75 (22.9)	22 (22.5)					
			0.82				
G	339 (51.7)	101 (51.5)		0.97			
A	317 (48.3)	95 (48.5)					
	Organ failure						
rs5743551	No	Yes					
AA	75 (26.0)	27 (22.7)					
AG	129 (44.8)	63 (52.9)		0.32	0.77	0.45–1.29	0.32
GG	84 (29.2)	29 (24.4)					
			0.08				
A	279 (48.4)	117 (49.2)		0.85			
G	297 (51.6)	121 (50.8)					
rs4833095							
GG	80 (27.7)	27 (22.5)					
AG	142 (49.1)	69 (57.5)		0.23	0.73	0.43–1.25	0.26
AA	67 (23.2)	24 (20.0)					
			0.81				
G	302 (52.3)	123 (51.3)		0.80			
A	276 (47.8)	117 (48.8)					

a For each outcome, genotype or allele counts are given with percentages in parentheses.

b Hardy-Weinberg equilibrium determined by exact test in survivors or in individuals without organ failure.

c Crude genotypic or allelic association with outcome determined by Chi square test.

d Adjusted association of genotype with outcome determined by logistic regression assuming a recessive model (G allele for each variant), adjusted for age, gender, pre-existing medical condition, and clinical presentation.

### rs5743551 and rs4833095 are not associated with Thai whole blood cytokine responses to a TLR1 agonist

**Figure 2:**
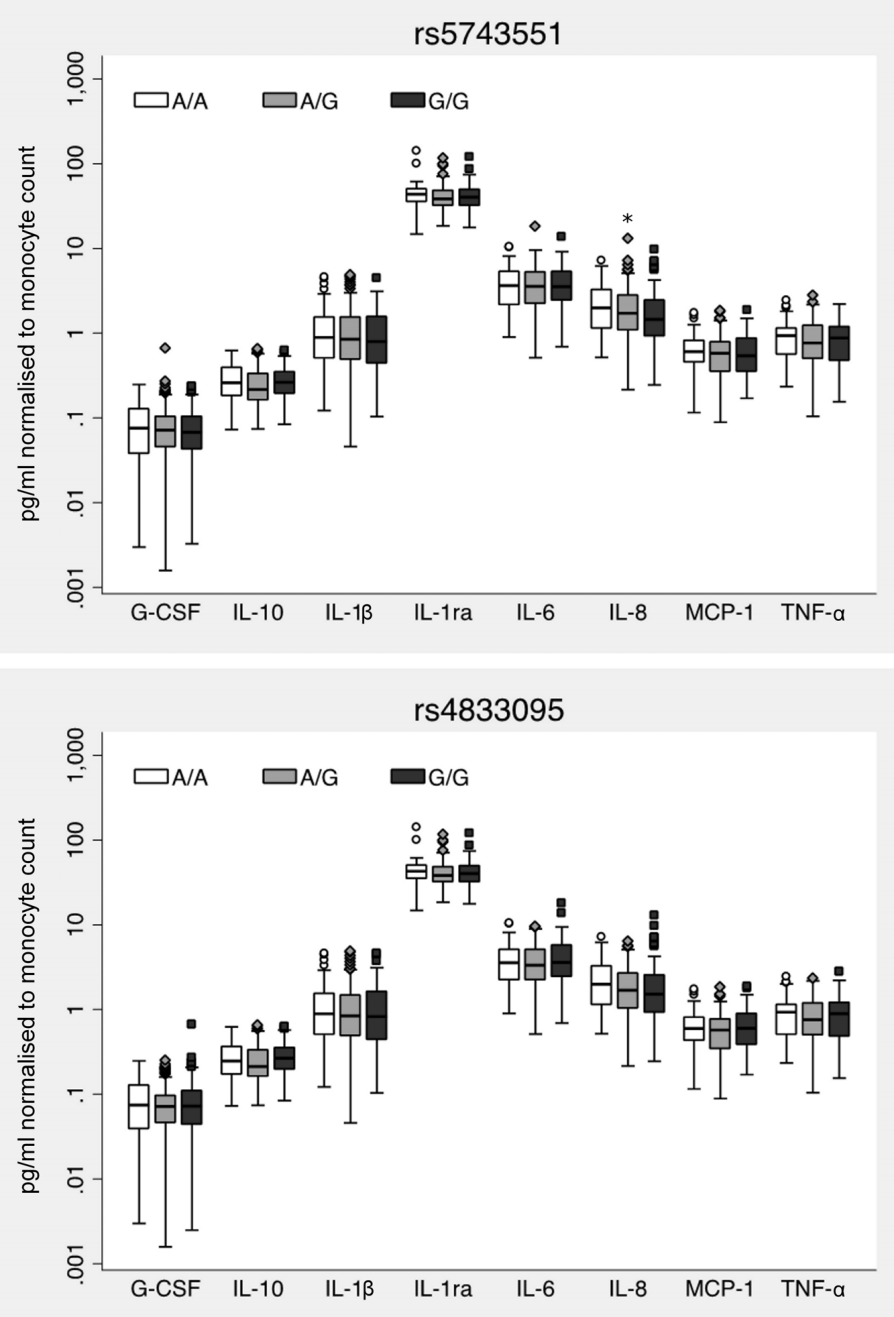
rs5743551 and rs4833095 are not associated with Thai whole blood cytokine responses to a TLR1 agonist. Whole blood was stimulated with Pam3CSK4 100/mL for six hours. Cytokine and chemokines were measured in plasma by multiplex bead assay, normalized to monocyte count, and log_10_ transformed for statistical analysis by linear regression, adjusting for age, gender, and batch. Boxes show the median and interquartile range; whiskers show upper and lower adjacent values. For rs5743551, N = 57 (AA), 147 (A/G), 96 (GG). For rs4833095, N = 57 (AA), 139 (A/G), 103 (GG). Cytokine responses were not significantly different by either genotype (P>0.05 for all comparisons).

As rs5743551 is associated with altered immune responses by whole blood in white American and African American healthy subjects [16], we tested whether a similar phenotype was evident in Thais. We stimulated whole blood from 300 healthy Thai subjects with Pam3CSK4, a specific TLR1 agonist. We observed a range of up to one log in cytokine production suggesting substantial inter-individual variation in the innate immune response to a TLR1 agonist. We genotyped rs5743551 and rs4833095 in these individuals and found that LD was again high (R^2^ = 0.91). Genotype and allele frequencies for these variants are given in Table 2. No variant deviated from Hardy-Weinberg equilibrium. In contrast to previous associations of rs5743551 genotype with altered Pam3CSK4-induced G-CSF, IL-1ra, IL-1β, IL-6, IL-8, IL-10, and TNF-α responses in white American subjects [16], we found only a modest reduction in IL-8 level (P = 0.03) but no significant alteration in any other cytokine for rs5743551 (Figure 2). We observed no rs4833095-dependent difference in inflammatory mediator production induced by Pam3CSK4 (Figure 2).

**Table 2:** *TLR1* variant genotype and allele frequencies in healthy Thai subjects.

Marker	Counts (%)	HWE^b^
		P
rs5743551		
AA	57 (19.0)	
AG	147 (49.0)	
GG	96 (32.0)	
		1.0
A	261 (43.5)	
G	339 (56.5)	
rs4833095		
GG	103 (34.5)	
AG	139 (46.5)	
AA	57 (19.1)	
		0.41
G	345 (57.7)	
A	253 (42.3)	
rs5743562		
AA	163 (54.3)	
AG	115 (38.3)	
GG	22 (7.3)	
		0.77
A	441 (73.5)	
G	159 (26.5)	
rs5743563		
TT	163 (54.3)	
TC	115 (38.3)	
CC	22 (7.3)	
		0.77
T	441 (73.5)	
C	159 (26.5)	
rs5743592		
TT	159 (53.7)	
TC	115 (38.9)	
CC	22 (7.4)	
		0.88
T	433 (0.73)	
C	159 (0.27)	
rs5743593		
TT	164 (54.7)	
TC	114 (38.0)	
CC	22 (7.3)	
		0.77
T	442 (73.7)	
C	158 (26.3)	
rs5743595		
TT	163 (54.3)	
TC	115 (38.3)	
CC	22 (7.3)	
		0.77
T	441 (73.5)	
C	159 (26.5)	
rs5743596		
CC	252 (84.0)	
CT	46 (15.3)	
TT	2 (0.7)	
		1.0
C	550 (0.92)	
T	50 (0.08)	
rs5743604		
TT	81 (27.1)	
TC	145 (48.5)	
CC	73 (24.4)	
		0.64
T	307 (51.3)	
C	291 (48.7)	

b Hardy-Weinberg equilibrium determined by exact test in all subjects.

### Other common *TLR1* variants are not associated with Thai whole blood cytokine responses to a TLR1 agonist

**Figure 3:**
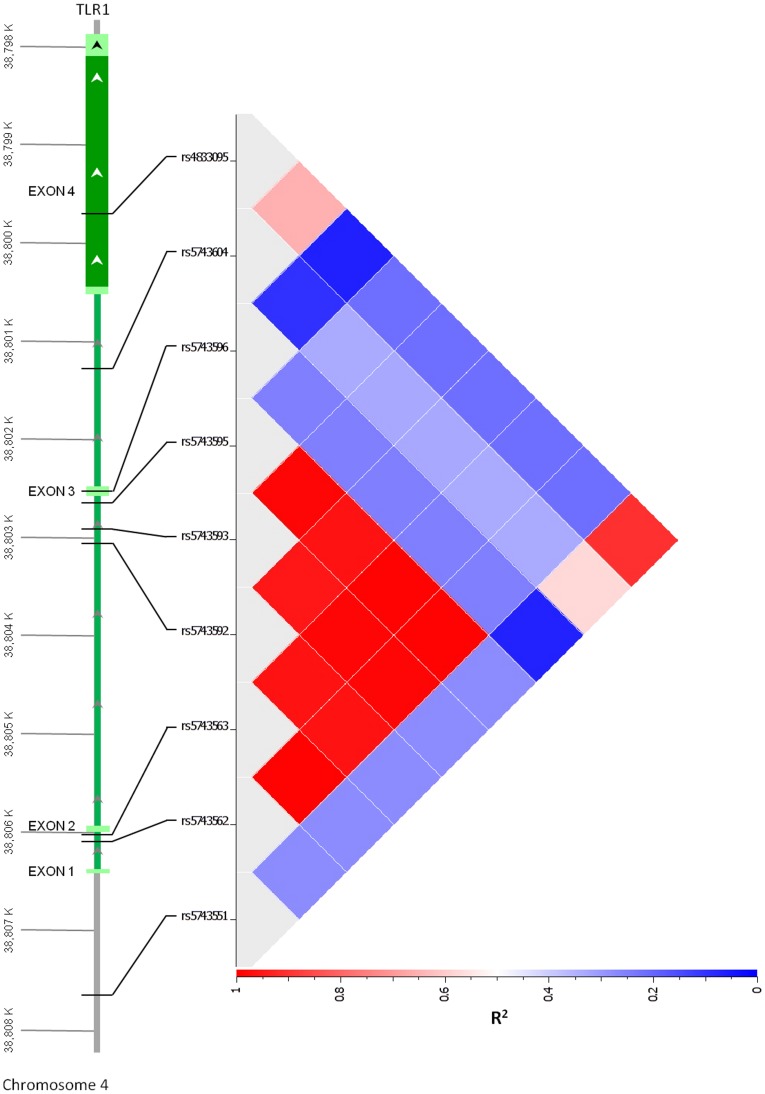
Linkage disequilibrium plot of common *TLR1* variants in Thais. Linkage disequilibrium (R^2^) for nine common *TLR1* variants in 300 Thai blood donors. Plot created using Golden Helix software.

As these common variants did not have an apparent functional association in our cohort, we then searched for other *TLR1* variants in Asian subjects that might underlie the variable innate immune response of Thai subjects to Pam3CSK4. We identified *TLR1* region variants as described in the methods and genotyped seven additional polymorphisms (rs5743562, rs5743563, rs5743592, rs5743593, rs5743595, rs5743596, and rs5743604). Genotype and allele frequencies are given in Table 2. Five of these variants (rs5743593, rs5743595, rs5743563, rs5743562, and rs5743592) were in very high LD (R^2^≥0.96) (Figure 3) so we selected one tag SNP from this bin (rs5743595) and the two other variants (rs5743604 and rs5743596) for analysis. However, we found no consistent association of any of the three polymorphisms with cytokine level induced by Pam3CSK4 (Table S2).

## Discussion

The results of this investigation show that common variation in *TLR1* in Thais is not associated with altered inflammatory responses to Pam3CSK4 in blood or with outcome from melioidosis, a common cause of sepsis in northeast Thailand. Our findings also confirm that the genetic architecture of functional *TLR1* variation is substantially different in southeast Asians compared to other global populations.

Sepsis remains a vexing syndrome to identify and treat [33], [34]. Targeting the innate immune response driven by activation of pathogen recognition sensors such as TLRs has been seen as a fruitful approach [35]. Indeed, numerous studies support the role of TLR pathway molecules in the pathogenesis of sepsis [36]. Only relatively recently, however, has TLR1 been carefully studied as an important regulator of the host response in sepsis, particularly in human disease. Our study of genetic variation in Thais with melioidosis offers an opportunity to examine the association of common *TLR1* variants with clinical outcome from one of the major Gram-negative causes of sepsis in northeast Thailand. Immunoassays in subjects recruited from the same population can provide corroborating functional evidence of inflammatory effects attributable to genotype. However, our data do not implicate known common genetic variants in *TLR1* in modulating the host response clinically or under experimental conditions.

In white Americans, rs5743551 is in moderate to high LD with the non-synonymous coding variants rs5743618 and rs4833095. All three variants (G, T, and G alleles, respectively) have been associated with greater inflammation or death from sepsis [16], [20], [21]. The rs5743618 T allele is also associated with susceptibility to leprosy [19], [24] but protection against tuberculosis and candidemia [37]–[39]. Yet, our findings suggest that in Thais, rs5743551 tags only rs4833095, the minor alleles of both polymorphisms are reversed compared to white Americans, and neither variant is associated with outcome from melioidosis. Although the G allele of rs5743618, encoding serine, has impaired cell surface trafficking and signaling [16], [17], [40], in Chinese and Vietnamese subjects this allele is very rare and, unlike in white American populations, is not tagged by rs5743551. We cannot exclude effects of rs5743618 in Thais, but the very low minor allele frequency in other southeast Asians argues against a significant explanatory effect of this genotype on clinical outcome. Furthermore, these data underscore the variability in *TLR1* genetic architecture across populations.

Despite the documented functional effects of rs5743618, there is a stronger association of the tag SNP rs5743551 G allele with death from sepsis than for the rs5743618 T allele in white North Americans [16], [20]. In white Americans with Gram-positive trauma-related sepsis, the rs5743551 G allele, but not the rs5743618 T allele, is associated with death [20]. These data argue in favor of additional functional *TLR1* variation tagged by rs5743551. rs4833095 is a possible candidate that is in fact associated with mortality in this Gram-positive trauma-related sepsis population [20]. The variant is also associated with susceptibility to infection. In a Brazilian cohort, the rs4833095 G allele (but not rs5743618) is associated with leprosy [31]. In addition, this allele is also associated with leprosy in Bangladeshi populations where the rs5743618 T allele occurs with a frequency of about 5% and the rs4833095 A allele is associated with placental malaria in Ghanaians where the rs5743618 G allele frequency is 2% [22], [23]. We have previously reported associations of rs5743351 and rs4833095 with susceptibility to melioidosis [14]. Yet, the mechanisms that modulate susceptibility to infection may well differ from those that regulate outcome once infection is established. For example, we have found that a common nonsense *TLR5* polymorphism, rs5744168, is strongly protective against death in this cohort, but is not associated with susceptibility to melioidosis [14], [15].

In addition to the absence of an association of rs4833095 with death from melioidosis in Thais, our present functional studies do not support an effect of rs4833095 on the host inflammatory response. Notably, the complete lack of effect on Pam3CSK4-induced cytokine release by rs4833095 in Thai subjects stands in contrast to recent work by Mikacenic et al [41]. Using a similar immuno-assay design, these investigators found an extremely strong effect of rs4833095 on IL-6 and TNF-α release induced by stimulation of whole blood from white subjects with Pam3CSK4. Yet, assays of HEK293 cells transfected with this variant (independent of rs5743618) have not shown altered NF-κB activation in response to Pam3CSK4 [16], [17]. Combined, the data suggest that in white North Americans, rs4833095 may not itself be functional but rather may tag a causative variant. In contrast, in Thais, there is no clear evidence of functionality, intrinsic or otherwise, attributable to rs4833095.

Our screening analysis of additional common *TLR1* variation did not reveal any other variants associated with altered inflammatory response to Pam3CSK4. Our approach was restricted to previously described variation, however, and it is clear that there is marked variation in the genetic architecture of *TLR1* across populations, possibly driven by infectious disease [24], [40], that may account for our negative results. We speculate that uncharacterized functional *TLR1* genetic variation in Asians remains to be discovered, and that this may regulate outcomes in sepsis. Although current genome wide association studies chips are characterized by a relative paucity of Asian variants, ongoing comprehensive sequencing efforts across a variety of populations are facilitating identification of potentially clinically relevant variants in relatively understudied populations [32].

While melioidosis is representative of Gram-negative sepsis, a prior study of *TLR1* polymorphisms in sepsis noted a relationship (in several cohorts) between frequency of Gram-positive infection and rs5743551 genotype [16]. Although TLR1 augments TLR2-mediated NF-κB activation by *B. pseudomallei* in vitro [25], suggesting a role for TLR1 signaling in melioidosis, it is conceivable that clinical associations of rs5743551 or rs4833095 might be limited to Gram-positive sepsis in Thais. Ongoing studies by our group are addressing this question.

Potential limitations to all genetic association studies include population stratification due to ethnic admixture and other unmeasured confounding. Previously, however, we have not observed substantial population stratification in our Sappasithiprasong cohort [14]. In addition, we cannot exclude the possibility that some severely ill patients died before enrolment into our study. Our melioidosis cohort is notable for being one of the few large populations of patients infected with a single Gram-negative etiology of sepsis and is further strengthened by the large scale immuno-assay study of healthy subjects from the same northeastern Thai population.

## Conclusion

In conclusion, in our cohort of Thai subjects with melioidosis we do not confirm the previously described associations in Spanish and white North American populations of *TLR1* genetic variation with outcomes from sepsis. We are also unable to identify functional effects of selected common *TLR1* variants in Thais. We confirm substantial differences in the genetic architecture of *TLR1* variation of Thais compared to other populations. These findings underscore the need for additional studies of *TLR1* and other innate immune genetic modulators of the inflammatory host response and determinants of sepsis in southeast Asian populations.

## Supporting Information

Table S1 **TLR1 variant genotype frequencies in selected global populations.**
(DOCX)

Table S2 **Minor allele frequencies and association of** ***TLR1*** **variants with cytokine induced by stimulation of whole blood with Pam3CSK4 in healthy Thai subjects.**
(DOCX)
