# Supplementary material for: Common TLR1 Genetic Variation Is Not Associated with Death from Melioidosis, a Common Cause of Sepsis in Rural Thailand
Source: PLoS One. 2014 Jan 2;9(1):e83285. doi: 10.1371/journal.pone.0083285 (PMC3879377; doi:10.1371/journal.pone.0083285)
Supplement: Table S1 — TLR1 variant genotype frequencies in selected global populations. (DOCX) [file pone.0083285.s001.docx]

Table S1. *TLR1* variant genotype frequencies in selected global populations

| Cohort | rs5743551 (-7202A/G) | | | | | | rs4833095 (742A/G or 743A/G) | | | | | | | | rs5743618 (1804G/T or 1805G/T) | | | | | | | |
| --- | --- | --- | --- | --- | --- | --- | --- | --- | --- | --- | --- | --- | --- | --- | --- | --- | --- | --- | --- | --- | --- | --- |
|  |  | |  | |  | |  | |  |  |  | |  | | |  | |  | |  | |  |
|  | AA | AG | | GG | |  | | AA | | AG | | GG | |  | | | GG | | GT | | TT | |
|  |  |  | |  | |  | |  | |  | |  | |  | | |  | |  | |  | |
| NORTH AMERICA |  |  | |  | |  | |  | |  | |  | |  | | |  | |  | |  | |
| Healthy white subjects, Seattle, USA [16] | 152  (55.3%) | 106  (38.5%) | | 17  (6.2%) | |  | |  | |  | |  | |  | | | 137  (49.8%) | | 108  (39.3%) | | 30  (10.9%) | |
| White sepsis patients, Vancouver, Canada [16] | 374  (52.6%) | 273  (38.4%) | | 64  (9.0%) | |  | |  | |  | |  | |  | | | 333  (48.5%) | | 253  (36.8%) | | 101  (14.7%) | |
| White trauma patients, Seattle, USA [20] | 823  (57.7%) | 517  (36.3%) | | 86  (6.0%) | |  | |  | |  | |  | |  | | | 706  (50.2%) | | 568  (40.4%) | | 132  (9.3%) | |
| Healthy white controls, USA [16] | 72  (43.9%) | 79  (48.2%) | | 13  (7.9%) | |  | |  | |  | |  | |  | | | 68  (40.7%) | | 84  (50.3%) | | 15  (8.9%) | |
| Utah residents with N. and W. European ancestry (CEU) [32] | 58  (68.2%) | 24  (28.2%) | | 3  (3.5%) | |  | | 58  (68.2%) | | 24  (28.2%) | | 3  (3.5%) | |  | | | 60  (70.6%) | | 21  (24.7%) | | 4  (4.7%) | |
| EUROPE |  |  | |  | |  | |  | |  | |  | |  | | |  | |  | |  | |
| Sepsis patients, Spain [21] | 86  (39.8%) | 96  (44.4%) | | 34  (15.7%) | |  | |  | |  | |  | |  | | | 67  (31.2%) | | 95  (45.0%) | | 49  (23.2%) | |
| SOUTH AMERICA |  |  | |  | |  | |  | |  | |  | |  | | |  | |  | |  | |
| Healthy controls, Baura, Brazil [31] |  |  | |  | |  | | 105  (28%) | | 174  (46%) | | 97  (26%) | |  | | | 55  (15%) | | 191  (50%) | | 133  (35%) | |
| AFRICA |  |  | |  | |  | |  | |  | |  | |  | | |  | |  | |  | |
| Healthy controls, The Gambia [24] |  |  | |  | |  | |  | |  | |  | |  | | | 0  (0.0%) | | 12  (3.4%) | | 337  (96.6%) | |
| Infants, S. Africa [30] |  |  | |  | |  | |  | |  | |  | |  | | | 3  (1.3%) | | 45  (20.0%) | | 176  (78.7%) | |
| ASIA |  |  | |  | |  | |  | |  | |  | |  | | |  | |  | |  | |
| Healthy controls, New Delhi, India [24] |  |  | |  | |  | |  | |  | |  | |  | | | 7  (2.9%) | | 48  (20.1%) | | 184  (77.0%) | |
| Leprosy patients, Nepal [29] |  |  | |  | |  | | 151  (18.1%) | | 405  (48.5%) | | 279  (33.4%) | |  | | | 9  (1.1%) | | 86  (10.7%) | | 706  (88.1%) | |
| Cord blood controls, Vietnam [17] |  |  | |  | |  | | 92  (23.6%) | | 208  (53.3%) | | 90  (23.1%) | |  | | | 0  (0.0%) | | 7  (1.9%) | | 368  (98.1%) | |
| Southern Han Chinese (CHS) [32] | 15  (15.0%) | 50  (50.0%) | | 35  (35.0%) | |  | | 15  (15.0%) | | 52  (52.0%) | | 33  (33.0%) | |  | | | 0  (0.0%) | | 3  (3.0%) | | 97  (97.0%) | |
| Blood donors and diabetics, Thailand [14] | 223  (23.5%) | 462  (48.7%) | | 263  (27.7%) | |  | | 215  (22.8%) | | 459  (48.6%) | | 271  (28.7%) | |  | | |  | |  | |  | |
