# Supplementary material for: Common TLR1 Genetic Variation Is Not Associated with Death from Melioidosis, a Common Cause of Sepsis in Rural Thailand
Source: PLoS One. 2014 Jan 2;9(1):e83285. doi: 10.1371/journal.pone.0083285 (PMC3879377; doi:10.1371/journal.pone.0083285)
Supplement: Table S2 — Minor allele frequencies and association of TLR1 variants with cytokine induced by stimulation of whole blood with Pam3CSK4 in healthy Thai subjects. (DOCX) [file pone.0083285.s002.docx]

Table S2. Minor allele frequencies and association of *TLR1* variants with cytokine induced by stimulation of whole blood with Pam3CSK4 in healthy Thai subjects

| SNP | MAF | P for association of genotype with cytokine level ^a^ | | | | | | | |
| --- | --- | --- | --- | --- | --- | --- | --- | --- | --- |
|  |  | G-CSF | IL-10 | IL-1β | IL-1ra | IL-6 | IL-8 | MCP-1 | TNF-α |
| rs5743604 | 0.49 | 0.75 | 0.55 | 0.65 | 0.42 | 0.83 | 0.16 | 0.87 | 0.56 |
| rs5743596 | 0.08 | 0.53 | 0.21 | 0.25 | **0.01** | 0.63 | 0.69 | 0.89 | 0.95 |
| rs5743595 | 0.27 | 0.29 | 0.31 | 0.86 | 0.10 | 0.09 | 0.06 | 0.74 | 0.07 |

^a^ Minor allele frequency

^b^ Association tested using linear regression with an additive model, adjusting for age, gender, and batch.
